# Three-Axis Vibration Isolation of a Full-Scale Magnetorheological Seat Suspension

**DOI:** 10.3390/mi15121417

**Published:** 2024-11-26

**Authors:** Young T. Choi, Norman M. Wereley, Gregory J. Hiemenz

**Affiliations:** 1 Department of Aerospace Engineering, University of Maryland, College Park, MD 20742, USA; wereley@umd.edu; 2 InnoVital Systems Inc., Calverton, MD 20705, USA; greg@innovitalsystems.com

**Keywords:** three-axis vibration isolation, aircraft vibration, seat suspension, magnetorheological

## Abstract

This study examines the three-axis vibration isolation capabilities of a full-scale magnetorheological (MR) seat suspension system utilizing experimental methods to assess performance under both single-axis and simultaneous three-axis input conditions. To achieve this, a semi-active MR seat damper was designed and manufactured to address excitations in all three axes. The damper effectiveness was tested experimentally for axial and lateral motions, focusing on dynamic stiffness and loss factor using an MTS machine. Prior to creating the full-scale MR seat suspension, a scaled-down version at one-third size was developed to verify the damper’s ability to effectively reduce vibrations in response to practical excitation levels. Additionally, a narrow-band frequency-shaped semi-active control (NFSSC) algorithm was developed to optimize vibration suppression. Ultimately, a full-scale MR seat suspension was assembled and tested with a 50th percentile male dummy, and comprehensive three-axis vibration isolation tests were conducted on a hydraulic multi-axis simulation table (MAST) for both individual inputs over a frequency range up to 200 Hz and for simultaneous multi-directional inputs. The experimental results demonstrated the effectiveness of the full-scale MR seat suspension in reducing seat vibrations.

## 1. Introduction

Excessive vibrations from ground and aerial vehicles can adversely affect the human body of both crew members and passengers. Short-term exposure to vibrations results in discomfort, inattention, distraction, and fatigue, which can degrade operational performance and situational awareness [1]. Moreover, prolonged exposure to whole-body vibrations may lead to serious health issues, such as back pain and injuries to the neck and spine [1,2]. Notably, vibrations up to 80 Hz can significantly impact various parts of the human body [3,4]. Specifically, the resonant frequencies for the neck and lumbar regions fall between 2.5 and 5.5 Hz, while the head and shoulder regions resonate between 20 and 30 Hz [4]. Extended exposure to these vibrations can lead to chronic musculoskeletal stress in affected areas, potentially resulting in permanent damage [5]. Given these concerns, the design of seat suspensions has become critical for both commercial and military vehicles.

Seat suspensions are configured between the occupant and the vibration source (i.e., floor motion), and designed to reduce the vibration transmitted to the occupant. Conventional passive seat suspensions have only a cushion pad and/or use passive hydraulic dampers. These conventional passive seat suspensions can cope with only either a small vibration input or a specific vibration excitation input frequency. For achieving vibration reduction for a larger vibration input and wider operating frequency range, active seat suspensions are developed. But active seat suspensions need an external power source for operation and thus become more complex, more expensive, and less stable than passive seat suspensions. For a compromise between passive and active seat suspensions, semi-active seat suspensions are proposed. In this study, a promising semi-active seat suspension, which uses magnetorheological (MR) fluid-based seat dampers as actuators, is investigated because MR fluid-based actuators can offer continuously controllable and intrinsically stable damper force, fast response time, and less power consumption.

Choi et al. [6,7,8] developed both electrorheological (ER) and MR seat suspensions for commercial ground vehicles and experimentally observed that ER and MR seat suspensions could successfully suppress the vibration by using skyhook and sliding mode control algorithms. Park and Jeon [9] formulated a Lyapunov-based robust control algorithm to compensate for actuator time delay and applied this robust control algorithm to an MR seat suspension. They analytically found that the proposed robust control algorithm could achieve good vibration suppression performance in the MR seat suspension with time delay. McManus et al. [10] constructed an MR seat suspension for heavy road vehicles by retrofitting a commercial hydraulic seat damper with an MR seat damper. They experimentally showed that the MR seat suspension could reduce the frequency and severity of end-stop impacts and attenuate whole-body vibration exposure levels. Choi and Wereley [11] analytically evaluated the biodynamic responses of an MR seat suspension to both sinusoidal vibration and shock crash loadings, and compared these results with passive hydraulic seat suspensions. Hiemenz et al. [12] experimentally explored the use of MR seat dampers in a helicopter crew seat suspension to enhance occupant comfort and reduce health issues. In addition, they proposed a semi-active simple load-limiting control algorithm for an MR seat suspension to enhance the helicopter crash survivability [13]. Sapinski et al. [14,15,16] experimentally observed the effectiveness of an MR seat suspension with a fuzzy control and simple semi-active controls under sinusoidal and shock transient inputs. Segla et al. [17] analytically evaluated the vibration reduction performance of an MR seat suspension for a bucket-wheel excavator. For better vibration reduction performance, they used a modified skyhook control whose control parameters were optimized by applying genetic algorithms. Yao et al. [18] formulated a controller for an MR seat suspension so as to deal with the actuator saturation and system parameter uncertainties. Li et al. [19] developed an MR elastomer (MRE) isolator for a seat suspension and experimentally evaluated the characteristics of the MRE isolator. In addition, they analytically showed the feasibility of an MRE-based seat suspension. Several research groups [20,21,22,23,24] developed scissor-mechanism types of seat suspensions using an MR damper and experimentally showed that the MR seat suspension could improve the vibration isolation performance compared to the passive seat suspension.

Most of the research reviewed above has concentrated on vibration suppression using a magnetorheological (MR) seat suspension only in the vertical direction. This focus is largely due to the fact that vertical excitation vibrations are prevalent and/or dominant in typical scenarios, and most MR seat dampers are capable of producing a controllable damper force along only one axis. However, in practical situations, occupants are often exposed to excitation inputs in all three axes. Moreover, experimental evaluations of the vibration isolation provided by MR seat suspensions have mostly been limited to a relatively low-frequency range, typically below 30 Hz. This limitation occurs because most experimental setups have been configured as single-degree-of-freedom (DOF) seat suspension systems with resonant frequencies under 10 Hz.

The objective of this study is to experimentally assess the three-axis vibration isolation performance of a full-scale MR seat suspension under both individual and simultaneous inputs in all three axes. To facilitate this, a semi-active MR seat damper was designed and manufactured that handle excitation inputs across all three axes. Damper performance, in terms of dynamic stiffness and loss factor, was evaluated experimentally for both axial and lateral axes using an MTS machine.

Before building the full-scale MR seat suspension, a 1/3 scale model was constructed to verify whether the newly manufactured MR seat damper could adequately reduce seat vibrations at practical excitation levels. In order to improve vibration suppression, a narrow-band frequency-shaped semi-active control (NFSSC) algorithm was implemented. Ultimately, a full-scale MR seat suspension was created, and three-axis vibration isolation tests were performed using a hydraulic multi-axis simulation table (MAST), employing a 50th percentile male dummy for both individual and simultaneous three-axis directional inputs.

## 2. Multi-Axis MR Seat Dampers for Seat Suspensions

**Figure 1:**
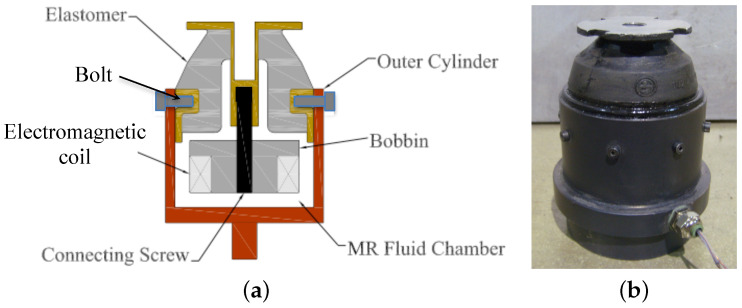
The multi-axis magnetorheological (MR) seat damper can be applied to either ground or air vehicle seat suspensions. (**a**) Schematic diagram and (**b**) fabricated seat damper.

Figure 1 is a schematic of the multi-axis MR seat damper that can be deployed either as a ground or air vehicle seat suspension. The electromagnetic coil is wrapped around the bobbin and the outer cylinder serves as a magnetic flux return. The bobbin is attached to the top elastomer through the connecting screw. When current input is applied to the electromagnetic coil, the magnetic density is concentrated at the bottom and radial sides of the bobbin. As a result, this MR seat damper can produce a resisting MR yield force in both the axial (i.e., vertical) and lateral (i.e., radial) directions. More details on the design and operating principles of this MR seat damper can be found in [25,26]. Figure 1b is a photograph of the multi-axis MR seat damper fabricated in this study. The height and outer diameter of the MR seat damper are 77 mm (3.0 in) and 60 mm (2.4 in), respectively. The bobbin and the outer cylinder were machined from 12L14 carbon steel and 26 gage copper wire was wound around the bobbin. There were 310 turns of the wire in the completed winding. The electrical resistance of the MR seat damper is 4.5 Ω. The weight of the MR seat damper is 709 g including MR fluid (in this case, Lord Corp MRF-132DG, Cary, NC, USA).

To assess whether the MR seat damper can handle multi-axis excitation inputs, the dynamic stiffness and loss angle of the MR seat damper were evaluated in both axial and lateral directions.

For a sinusoidal excitation displacement, $x_{e}\left(t\right)=X_{e0}\sin\left(\omega_{e}t\right)$, the transmitted damper force, through the MR seat damper, is given by

(1) $$F_{T}\left(t\right)=F_{T0}\sin\left(\omega_{e}t+\phi \right)$$

Here, $X_{e0}$ is the excitation displacement amplitude and $F_{T0}$ is the transmitted force amplitude. Here, $\omega_{e}$ is the excitation frequency and ϕ is the phase lead with respect to the excitation displacement. Then, a complex stiffness can be defined

(2) $$\begin{array}{ccc} K(\omega ) & = & \frac{F[F_{T}\left(t\right)]}{F[x(t)]}\;at\;\omega =\omega_{e} \end{array}$$

(3) $$\begin{array}{ccc} & = & K_{s}\left(\omega \right)+jK_{l}\left(\omega \right) \end{array}$$

Here, ω is the frequency and F[·] is the Fourier transform operator. Here, $K_{s}\left(\omega \right)$ is the storage stiffness and $K_{l}\left(\omega \right)$ is the loss stiffness. Finally, the dynamic stiffness, $K_{d}\left(\omega \right)$ and loss angle, $\phi_{a}\left(\omega \right)$ can be determined by

(4) $$K_{d}\left(\omega \right)=\sqrt{K_{s}{\left(\omega \right)}^{2}+K_{l}{\left(\omega \right)}^{2}}$$

(5) $$\phi_{a}\left(\omega \right)=\tan^{-1}\left(\frac{K_{l}\left(\omega \right)}{K_{s}\left(\omega \right)}\right)$$

**Figure 2:**
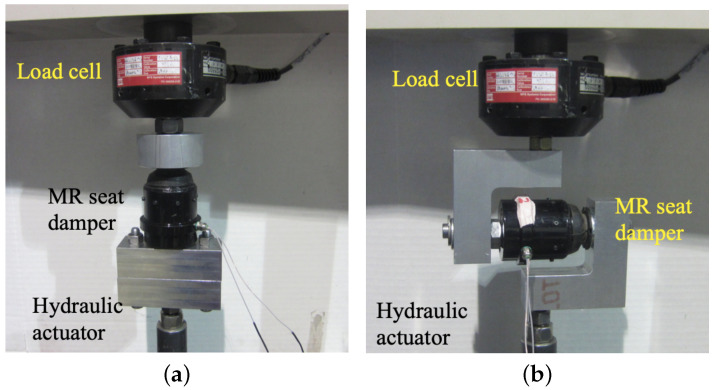
Experimental setup used to measure the damping performance of the single MR seat damper on an MTS machine. (**a**) Axial direction and (**b**) lateral direction.

Figure 2 is a photograph of the experimental setup on an MTS machine that was used when measuring the damping performance of the single MR seat damper. For both axial and lateral directions, testing was conducted using the same MTS machine. In these tests, the MR seat damper was initially compressed in the axial direction by 2 mm to simulate static deflection due to the occupant mass. A sinusoidal excitation input was chosen and its excitation frequency was changed from 1 Hz to 20 Hz with a constant amplitude of ±1.0 mm. The applied current input was varied over the range from 0 to 3 A.

**Figure 3:**
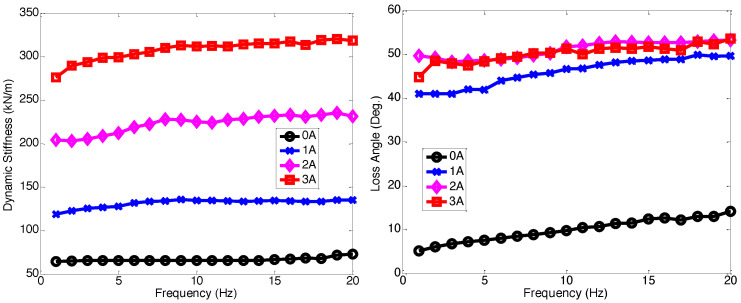
Axial dynamic stiffness and loss angle of the multi-axis MR seat damper under ±1.0 mm excitation displacement. Note that the initial axial compression was 2 mm.

Figure 3 presents the axial dynamic stiffness and loss angle of the multi-axis MR seat damper. As seen in this figure, the dynamic stiffness and loss angle increased, as the frequency increased. But in response to the applied current input, the dynamic stiffness and loss angle significantly increased. Compared to the zero-field (i.e., 0 A) input case, the 3 A current input case shows about four times larger dynamic stiffness and loss angle. This implies that when the current is applied, the stiffness of the MR seat damper becomes hard and its damping becomes big. Generally speaking, hard stiffness and high damping are good for the vibration reduction in a system at an axial resonant frequency.

**Figure 4:**
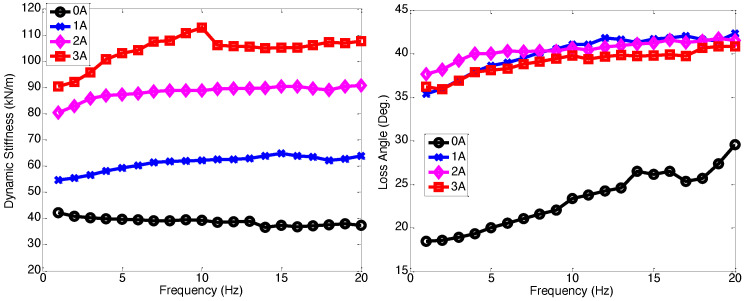
Lateral dynamic stiffness and loss angle of the multi-axis MR seat damper under ±1.0 mm excitation displacement. Note that the initial axial compression was 2 mm.

Figure 4 presents the lateral dynamic stiffness and the loss angle of the multi-axis MR seat damper. Similar to the axial excitation case, the lateral dynamic stiffness and loss angle also increased as frequency increased. However, in comparison to the axial excitation case, the increments in lateral dynamic stiffness and loss angle for the applied current input were smaller, but the MR seat damper still provided twice the dynamic stiffness and loss angle of the zero-field input case. This demonstrates that the MR seat damper has sufficient capability to effectively reduce seat vibration in the lateral directions. On the other hand, the loss angle, as seen in Figure 3 and Figure 4, did not exhibit a significant increase or even decrease at higher current inputs, which was a different trend compared to the dynamic stiffness. This phenomenon was due to the fact that the increases in either the storage stiffness or the loss stiffness, or both, can lead to an increase in the dynamic stiffness as seen in Equation (4). But if the loss stiffness does not increase as much as the storage stiffness, the loss angle can remain constant or even decrease since the loss angle is the inverse tangent of the ratio of the loss stiffness to the storage stiffness as seen in Equation (5).

## 3. Scaled-Down MR Seat Suspension Using an MR Seat Damper

Before we applied the MR seat dampers to a full-scale seat suspension, a 1/3 scale MR seat suspension was constructed and seat vibration isolation testing was conducted in the vertical (i.e., axial) direction to ensure that the MR seat suspension was capable of sufficiently reducing the seat vibration in response to practical excitation levels.

### 3.1. Testing Setup

**Figure 5:**
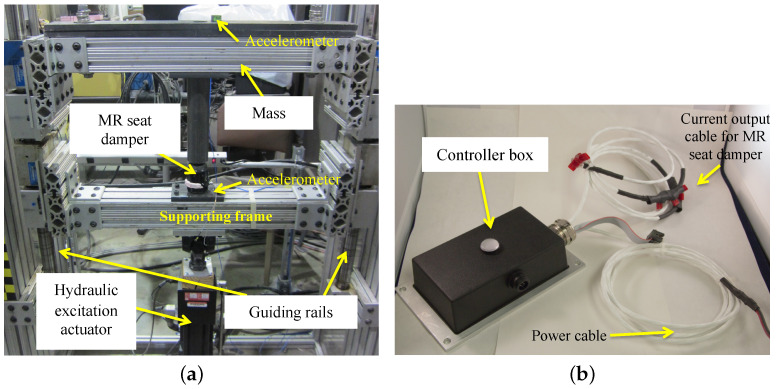
The single-degree-of-freedom (DOF) testing stand for the 1/3 scale MR seat suspension for the axial (i.e., vertical) direction: (**a**) test stand, (**b**) controller box.

Figure 5 presents the photograph of the single DOF testing stand for the 1/3rd scale-down MR seat suspension for the axial direction. The MR seat damper is placed between the mass and the hydraulic excitation actuator and the mass linearly moves along with the guiding rails. The mass is chosen to be equal to 1/3rd of the full-scale system mass because the full-scale MR seat suspension has three MR seat dampers. In this study, two different masses of 65 and 95 lbs were chosen for the vibration isolation tests. The hydraulic excitation actuator provides the excitation input displacement or acceleration. The accelerations of the excitation input and the mass were measured by the capacitive accelerometers because of higher sensing sensitivity. To supply current input to the MR seat damper, an in-house controller box (see Figure 5b) was used. The in-house controller box has a digital signal processor (DSP) chip that can be programmed to execute a desired vibration control algorithm. In addition, the controller box has a three-axis acceleration measurement capability and can be operated using a 28 V battery so that it can be implemented in a vehicle. Thus, this controller box determines the control current supplied by the MR damper based on acceleration measured using the on-board multi-axis accelerometer, without using any other external sensors or current amplifiers. The controller box was installed on the supporting frame to measure the acceleration excitation and supplied the control current to the MR seat damper during testing. Note that the capacitive accelerometers (see Figure 5a) were used only to evaluate the vibration isolation performance of the MR seat suspension, and were not used for feedback control.

### 3.2. Vibration Control Algorithm

In order to efficiently reduce the vibration of the MR seat suspension, a narrow-band frequency-shaped semi-active control (NFSSC) algorithm [27] was used. In a departure from classical semi-active control algorithms, this NFSSC algorithm was designed without resorting to an active vibration control algorithm superposed by the energy dissipation constraint (called “clipping control”). In addition, the NFSSC algorithm requires neither an accurate damper model nor system identification of damper model parameters. Also, the NFSSC algorithm can implemented using fewer sensors, both reducing cost and complexity.

**Figure 6:**
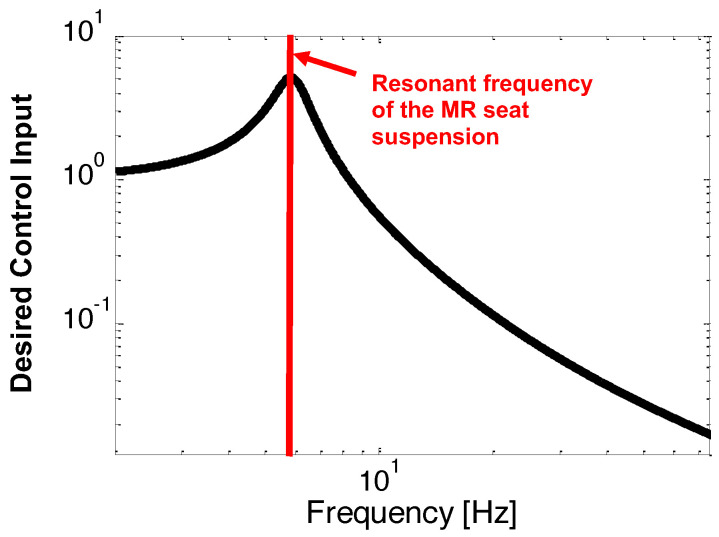
Desired control input shape of the narrow-band frequency-shaped semi-active control (NFSSC) algorithm in the frequency domain.

The NFSSC algorithm will apply a larger current input to the MR seat damper when the dominant frequency of the excitation input is close to the resonant frequency of the MR seat suspension for achieving high damping. But in frequencies other than the resonant frequency area, a minimized current input will be applied. Figure 6 depicts the desired control input shape of the NFSSC algorithm in the frequency domain. In this case, the desired frequency shaping of the control input shown in Figure 6 can be exactly matched to the frequency response behavior of the dynamics of a single DOF vibration isolation system as follows [28]:

(6) $$|\frac{z_{nout}\left(\omega \right)}{z(\omega )}|=\sqrt{\frac{1+4\zeta_{na}^{2}\frac{\omega^{2}}{\omega_{na}^{2}}}{(1-\frac{\omega^{2}}{\omega_{na}^{2}}{)}^{2}+4\zeta_{na}^{2}\frac{\omega^{2}}{\omega_{na}^{2}}}}$$

Here, $\omega_{na}$ and $\zeta_{na}$ are the resonant frequency and damping ratio of the single DOF vibration isolation system, respectively. Also, $z_{nout}$ is the output displacement of the single DOF vibration isolation system under the excitation displacement input, *z*. Note that the resonant frequency, $\omega_{na}$ of the single DOF vibration isolation system will be intentionally set to be close to the resonant frequency, $\omega_{n}$ of the MR seat suspension. The corresponding governing equation of the single DOF vibration isolation system given in Equation (6) is obtained as follows:

(7) $${\ddot{z}}_{nout}\left(t\right)=-\omega_{na}^{2}\left[z_{nout}\left(t\right)-z\left(t\right)\right]-2\zeta_{na}\omega_{na}\left[{\dot{z}}_{nout}\left(t\right)-\dot{z}\left(t\right)\right]$$

As seen in Figure 6, the output displacement, $z_{nout}$, will increase around the resonant frequency, $\omega_{n}$, of the MR seat suspension. If the control current input is determined to be proportional to the amplitude given in Equation (6), a larger current input will be obtained only in the narrow-band frequency range where it corresponds to the resonant frequency area of the single DOF vibration isolation system given in Equation (6). Finally, the control current input of the NFSSC algorithm is found as follows:

(8) $$i\left(t\right)=\left\{\begin{array}{cc} k_{n}|\frac{{\ddot{z}}_{nout}\left(t\right)}{\ddot{z}\left(t\right)}| & \mathrm{if}\;i\leq i_{max}\\ i_{max} & \mathrm{if}\;i>i_{max} \end{array}\right.$$

Here, $k_{n}$ is the control gain of the NFSSC algorithm. Note that the control current input given in Equation (8) can be also implemented using the ratio of the output displacement to the excitation displacement input. But in this study, the acceleration was used in Equation (8) because the acceleration could be measured more easily than displacement for this MR seat suspension implementation.

To calculate the control input given in Equation (8) in real time, we need to solve Equation (7) in real time. For a simple implementation, Equation (8) was discretized using the Euler method as follows:

(9) $$\begin{array}{c} z_{nout}\left(n\right)=\left[2-2\zeta_{na}\omega_{na}h\right]z_{nout}\left(n-1\right)-\left[1+\omega_{na}^{2}h^{2}-2\zeta_{na}\omega_{na}h\right]z_{nout}\left(n-2\right)\\ \;+2\zeta_{na}\omega_{na}hz\left(n-1\right)+\left[\omega_{na}^{2}h^{2}-2\zeta_{na}\omega_{na}h\right]z\left(n-2\right) \end{array}$$

Here, *h* is the time step of the calculation. In this study, the time step was chosen to be *h* = 0.5 ms. Using Equation (9), the final control current input, i(n) of the NFSSC is given by

(10) $$i\left(n\right)=\left\{\begin{array}{cc} k_{n}|\frac{z_{nout}\left(n\right)-2z_{nout}\left(n-1\right)+z_{nout}\left(n-2\right)}{z(n)-2z(n-1)+z(n-2)+\alpha }| & \mathrm{if}\;i\leq i_{max}\\ i_{max} & \mathrm{if}\;i>i_{max} \end{array}\right.$$

Here, α is the constant number that was used to prevent the denominator of Equation (10) becomes zero.

### 3.3. Experimental Results

**Figure 7:**
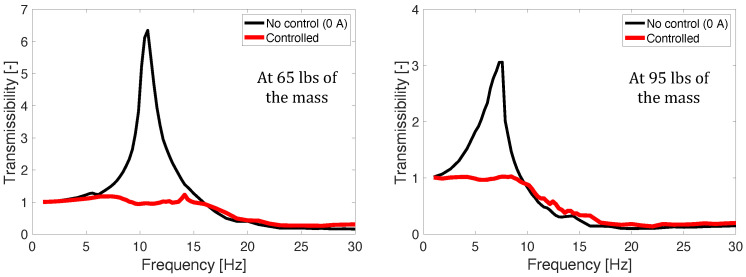
Transmissibility of the 1/3rd scale MR seat suspension for the axial direction (excitation ±0.1 g).

Figure 7 presents the transmissibility of the 1/3 scale MR seat suspension for the axial direction. In this case, the sinusoidal excitation input was swept from 1 to 30 Hz with a constant acceleration level of ±0.1 g. Here, $g=9.82\;m/s^{2}$. Note that this vibration acceleration level was chosen because it is a typical vibration level measured for seat suspensions in military propeller aircraft [2]. The control gain and the constant number of the NFSSC algorithm for the 95 lbs mass case were chosen to be $k_{n}$ = 20 and α = 1.5 g, respectively. For the 65 lbs mass case, these were chosen to be $k_{n}$ = 9 and α = 1.5 g, respectively. For all mass cases, the resonant frequency and damping ratio of the NFSSC algorithm were chosen to be $\omega_{na}$ = 6.5 Hz and $\zeta_{na}$ = 0.1, respectively. The maximum control current input, $i_{max}$, was limited to 2 A by the consideration of the current output capability of the in-house controller box because three MR seat dampers will be used for a full-scale MR seat suspension and they should be activated by one controller box (maximum current output capability was 6 A). In order to improve vibration isolation performance in the high-frequency range, the measured acceleration fed into Equation (10) was filtered by a low-pass filter with the cutoff frequency of 20 Hz, and then, the current input below 0.3 A was made to be zero. In this figure, the thin solid line stands for no control case (i.e., 0 A case) and the thick solid line implies the results controlled by the NFSSC algorithm. As seen in this figure, the MR seat damper using the NFSSC algorithm effectively reduces the vibration of the MR seat suspension for these the practical excitation levels. In addition, for both occupant masses, the MR seat damper using the NFSSC algorithm exhibited good seat vibration suppression.

## 4. Full-Scale Seat Suspension Using Multi-Axis MR Seat Dampers

**Figure 8:**
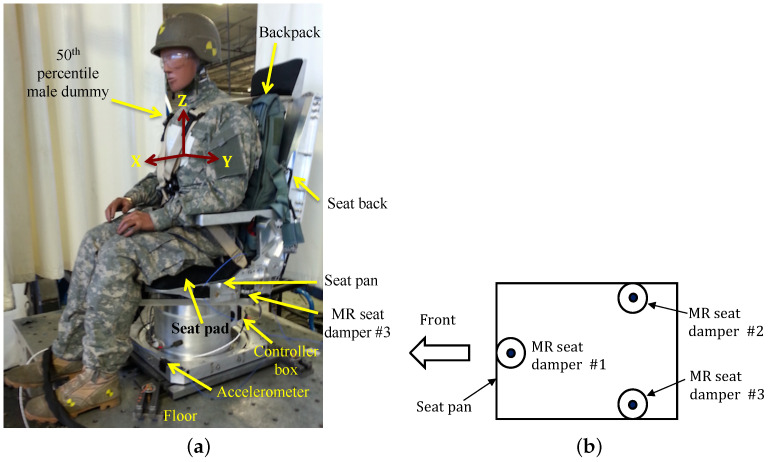
Test configuration of the full-scale MR seat suspension: (**a**) full-scale MR seat suspension, (**b**) MR seat damper configuration (top view).

Figure 8 shows the test setup of a full-scale MR seat suspension. The seat base was rigidly attached to the floor. The seat cushion was placed on the seat pan and the dummy was then seated on top of the cushion. The dummy was then tightly fastened to the seat by the seat belt to minimize unwanted dynamic impacts between the dummy and the seat. In this case, the 50th percentile male dummy was chosen and its total mass including the backpack and the sprung seat mass was 215 lbs. Three MR seat dampers were installed below the seat pan (see Figure 8b). These three MR seat dampers were electronically connected to the single controller box. The seat can accommodate three (*x*, *y*, *z* axes) translational motions. Here, the *x*-axis (i.e., fore-and-aft direction) is set by the normal direction to the face of the dummy pointing outward. The *y*-axis is the lateral direction and the *z*-axis is the vertical direction. For three-axis acceleration measurements, three-axis accelerometers were mounted on the floor, the seat pan, the seat pad, and the seat back. Note that these acceleration measurements were used only for the vibration isolation evaluation purpose, not for the vibration control purpose. For vibration isolation testing, the MR seat suspension was mounted on a hydraulic MAST and the floor was vibrated in the three-axis direction either individually or simultaneously. In this study, two different excitation inputs were used: the first is the sinusoidal input with a constant acceleration level of ±0.1 g and the second is representative seat transient input. The controller box was installed at the backside of the seat base and determined a control current input based on the three-axis excitation inputs measured by its own three-axis accelerometer.

The control current inputs ($i_{x}$, $i_{y}$, and $i_{z}$) in each direction of the NFSSC algorithm are given as follows:

(11) $$\mathrm{For}\;x-\mathrm{axis}\;\mathrm{direction}:\;i_{x}\left(n\right)=\left\{\begin{array}{cc} k_{n,x}|\frac{y_{out,x}\left(n\right)-2y_{out,x}\left(n-1\right)+y_{out,x}\left(n-2\right)}{y_{x}\left(n\right)-2y_{x}\left(n-1\right)+y_{x}\left(n-2\right)+\alpha_{x}}| & \mathrm{if}\;i_{x}\leq i_{max}\\ 0 & \mathrm{if}\;i_{x}\leq 0.3A\\ i_{max} & \mathrm{if}\;i_{x}>i_{max} \end{array}\right.$$

(12) $$\mathrm{For}\;y-\mathrm{axis}\;\mathrm{direction}:\;i_{y}\left(n\right)=\left\{\begin{array}{cc} k_{n,y}|\frac{y_{out,y}\left(n\right)-2y_{out,y}\left(n-1\right)+y_{out,y}\left(n-2\right)}{y_{y}\left(n\right)-2y_{y}\left(n-1\right)+y_{y}\left(n-2\right)+\alpha_{y}}| & \mathrm{if}\;i_{y}\leq i_{max}\\ 0 & \mathrm{if}\;i_{y}\leq 0.3A\\ i_{max} & \mathrm{if}\;i_{y}>i_{max} \end{array}\right.$$

(13) $$\mathrm{For}\;z-\mathrm{axis}\;\mathrm{direction}:\;i_{z}\left(n\right)=\left\{\begin{array}{cc} k_{n,z}|\frac{y_{out,z}\left(n\right)-2y_{out,z}\left(n-1\right)+y_{out,z}\left(n-2\right)}{y_{z}\left(n\right)-2y_{z}\left(n-1\right)+y_{z}\left(n-2\right)+\alpha_{z}}| & \mathrm{if}\;i_{z}\leq i_{max}\\ 0 & \mathrm{if}\;i_{z}\leq 0.3A\\ i_{max} & \mathrm{if}\;i_{z}>i_{max} \end{array}\right.$$

In addition, the control gains of the NFSSC algorithm were chosen as follows:

(14) $$\begin{array}{cc} & \;\mathrm{For}\;x-\mathrm{axis}\;\mathrm{direction}:\;k_{n,x}\;=\;9,\;\alpha_{x}\;=\;1.5\;g,\;\omega_{na,x}\;=\;8\;\mathrm{Hz},\;\mathrm{and}\;\zeta_{na,x}\;=\;0.1 \end{array}$$

(15) $$\begin{array}{cc} & \mathrm{For}\;y-\mathrm{axis}\;\mathrm{direction}:\;k_{n,y}\;=\;9,\;\alpha_{y}\;=\;1.5\;g,\;\omega_{na,y}\;=\;6.5\;\mathrm{Hz},\;\mathrm{and}\;\zeta_{na,y}\;=\;0.1 \end{array}$$

(16) $$\begin{array}{cc} & \;\mathrm{For}\;z-\mathrm{axis}\;\mathrm{direction}:\;k_{n,z}\;=\;9,\;\alpha_{z}\;=\;1.5\;g,\;\omega_{na,z}\;=\;9\;\mathrm{Hz},\;\mathrm{and}\;\zeta_{na,z}\;=\;0.1 \end{array}$$

Finally, the control input of the NFSSC algorithm for the vibration control of the three-axis full-scale MR seat suspension was given as follows:

(17) $$i\left(n\right)=i_{x}\left(n\right)+i_{y}\left(n\right)+i_{z}\left(n\right)$$

The control input given in Equation (17) was applied to the MR seat dampers by using the controller box.

### 4.1. Testing Results Under Sinusoidal Excitation Input

**Figure 9:**
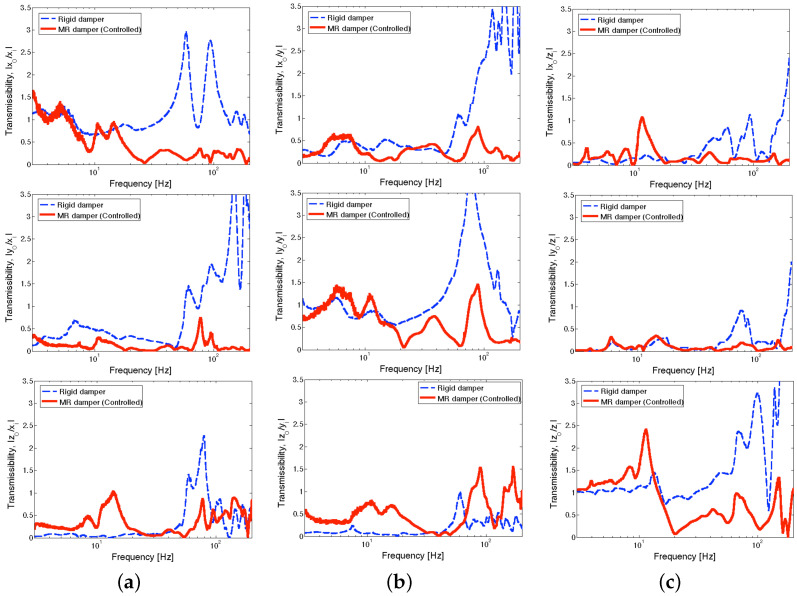
Three-axis transmissibility at the seat pan of the full-scale MR seat suspension using the NFSSC control algorithm for each directional excitation input (excitation level: ±0.1 g): (**a**) *x*-axis excitation input, (**b**) *y*-axis excitation input, (**c**) *z*-axis excitation input.

Figure 9 presents three-axis transmissibility at the seat pan of the full-scale MR seat suspension under the NFSSC algorithm for each directional excitation input. In this figure, the solid line stands for the controlled MR damper case and the dashed line stands for the rigid damper case. Note that in the rigid damper case, three MR seat dampers were replaced by three rigid aluminum blocks. In this study, the rigid damper case was used as the baseline performance level because this emulates the rigid connection of a seat to the airframe using only a seat pad for comfort, which has been used in military propeller aircraft. Here, the subscript “i” denotes excitation input, and the subscript “o” denotes the response. In this test, excitation acceleration input was applied to each axis individually for all three axes, and the transmissibility functions were determined using the input from one axis to the output accelerations from all three axes. Thus, a total of nine transmissibility functions were measured for the full-scale test case.

As seen in Figure 9, when the excitation input direction is the same as the output acceleration direction (for example, ${\ddot{x}}_{o}$/${\ddot{x}}_{i}$, ${\ddot{y}}_{o}$/${\ddot{y}}_{i}$, and ${\ddot{z}}_{o}$/${\ddot{z}}_{i}$), the transmissibility over the lower frequency range (i.e., below 20 Hz) becomes relatively large. On the other hand, the rigid damper case presents low transmissibility over the higher frequency range (i.e., after 30 Hz). Such higher transmissibility (greater than 1) in the higher frequency range may come from high-frequency resonant modes of the dummy and the seat. The controlled MR seat damper shows fairly low transmissibility over the relatively lower frequency range that is close to the performance of the rigid damper case. In addition, over the higher frequency range, the controlled MR seat damper achieved significant improvements in vibration isolation compared to the rigid damper case. This reduction in transmissibility demonstrates that the full-scale MR seat damper improves vibration isolation performance over the frequency range where the low-frequency resonant frequency of the seat suspension is dominant, that is, the NFSSC algorithm provides high damping (high current is supplied to the MR damper) over this frequency range as explained in Section 3.

For a quantative comparison of the transmissibility, the root mean square (RMS) transmissibility, $T_{RMS}$, was used, which is defined as

(18) $$T_{rms}=\sqrt{\frac{1}{\omega_{2}-\omega_{1}}\int_{\omega_{1}}^{\omega_{2}}T{\left(\omega \right)}^{2}d\omega }$$

**Figure 10:**
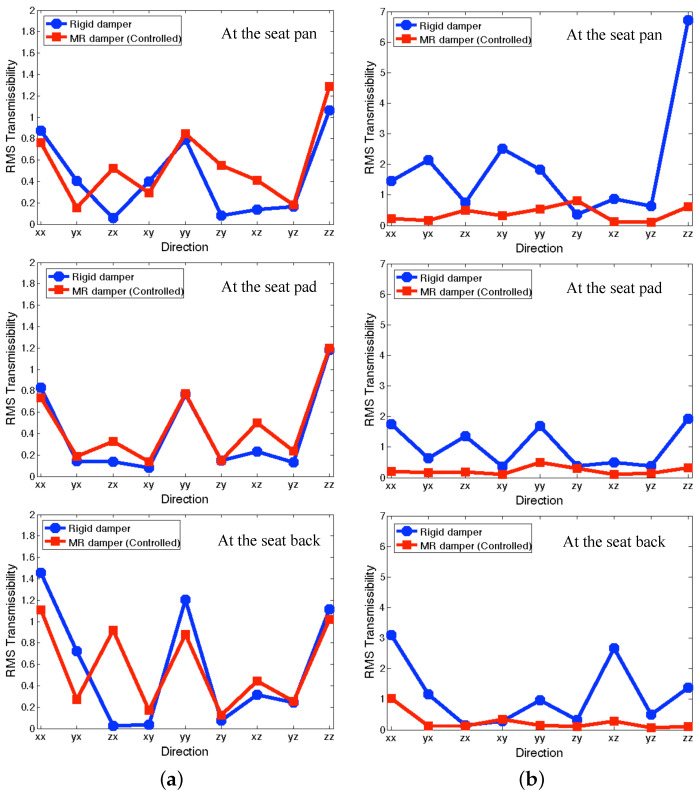
RMS transmissibility of the full-scale MR seat suspension using the NFSSC algorithm fo excitation input in each direction: (**a**) for the relatively low-frequency range (3–20 Hz), (**b**) for the higher frequency range (20–200 Hz).

Here, T(ω) is the transmissibility in the frequency domain, $\omega_{1}$ is the staring frequency, and $\omega_{2}$ is the ending frequency. A smaller value of RMS transmissibility implies better vibration isolation performance over the calculated frequency range. Figure 10 presents the RMS transmissibility of the full-scale MR seat suspension under the NFSSC algorithm for each directional excitation input. In the figure, the circle symbol stands for the rigid damper case and the rectangle symbol stands for the controlled MR seat damper case. In addition, the yz direction in this figure implies *y*-axis output to *z*-axis excitation input. This rule can be applied to the other cases, as in zx, xy, and yx, etc. Here, the frequency domain is divided into two regions: (a) a relatively low-frequency range of 3–20 Hz, (b) relatively high-frequency range of 20–200 Hz. As seen in this figure, for the relatively low-frequency range, the controlled MR seat damper case shows good vibration isolation performance at the seat pan similar to the rigid damper case. Similar vibration isolation performance was also observed at the positions of the seat pad and the seat back. But for the higher frequency range, the controlled MR seat damper case presents much better vibration isolation performance than the rigid damper case for most the input axes. On the other hand, at the positions of the seat pan and the seat pad, the largest RMS transmissibility occurs for the zz case. But at the position of the seat back, the largest RMS transmissibility occurs for the xx case. The reason is that *x*-axis output at the seat back also contains the front-and-back rocking motion. Another interesting observation is that the seat pad could not effectively reduce the relatively low-frequency dominant vibrations, but showed a certain level of vibration isolation for the high-frequency dominant vibrations. In addition, for the controlled MR seat damper case, the effect of the seat pad on the vibration suppression performance is insignificant.

### 4.2. Test Results Under Representative Seat Transient Inputs

**Figure 11:**
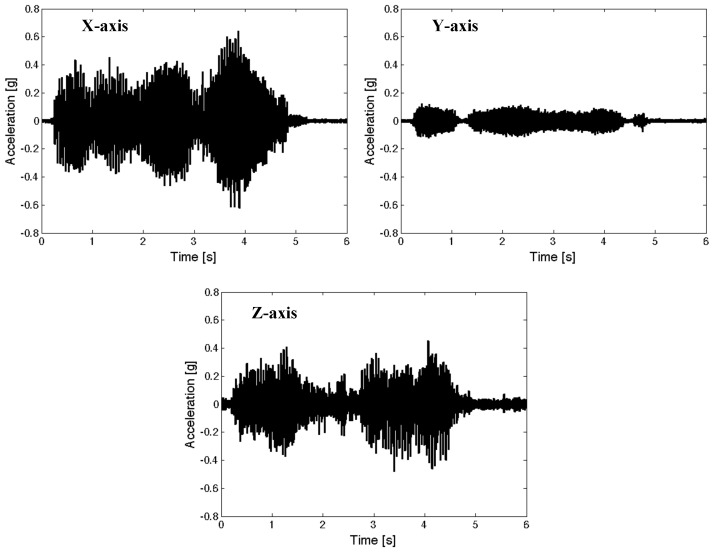
Measured time profiles of representative transient inputs for seat suspensions in a military propeller aircraft [29].

Figure 11 presents the measured time profiles of representative transient inputs for seat suspensions. These representative transient inputs were chosen from the floor excitations of seat suspensions in military propeller aircraft [29]. The representative transient excitation inputs have a frequency component of 0–80 Hz, but their dominant magnitudes were located at 18.5 and 75 Hz. In this study, the measured RMS values of representative transient excitation inputs were 0.171 g for the *x*-axis input, 0.043 g for *y*-axis input, and 0.101 g for *z*-axis input. Note that the transient input for the *x*-axis is greater than that for the *z*-axis for this military propeller aircraft.

**Figure 12:**
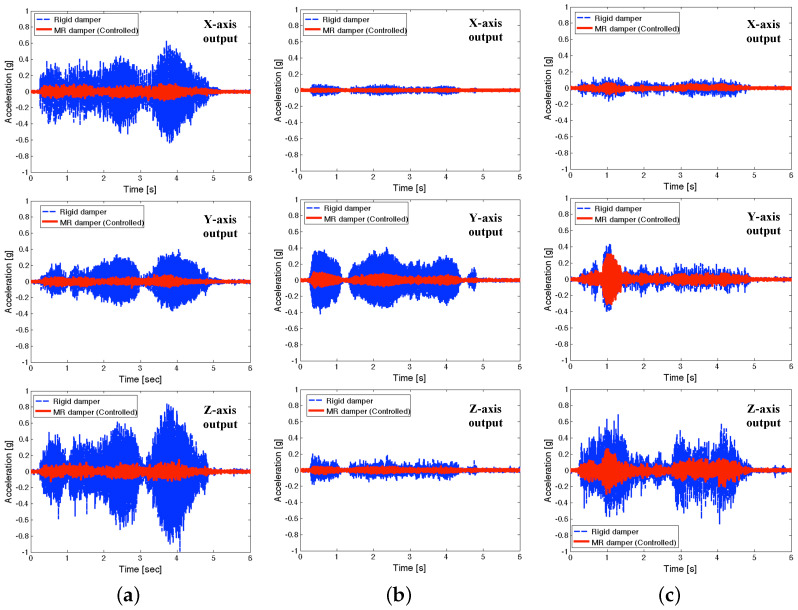
Measured time responses at the seat pan of the full-scale MR seat suspension using the NFSSC algorithm for each directional representative excitation input (**a**) for *x*-axis excitation input, (**b**) for *y*-axis excitation input, and (**c**) for *z*-axis excitation input.

Figure 12 presents the measured time responses at the seat pan of the full-scale MR seat suspension under the NFSSC algorithm for each directional representative excitation input. In this figure, the dashed line stands for the rigid damper case and the solid line stands for the controlled MR seat damper case. As seen in this figure, the *x*-axis excitation input case shows more severe transient acceleration outputs than other axes excitation input cases. In addition, for the *x*-axis transient input case, *z*-axis acceleration output is bigger than *x*-axis acceleration output. As seen in this figure, the controlled MR seat damper case shows much better vibration isolation performance at the position of the seat pan than the rigid damper case for each representative transient input for all three axes.

**Figure 13:**
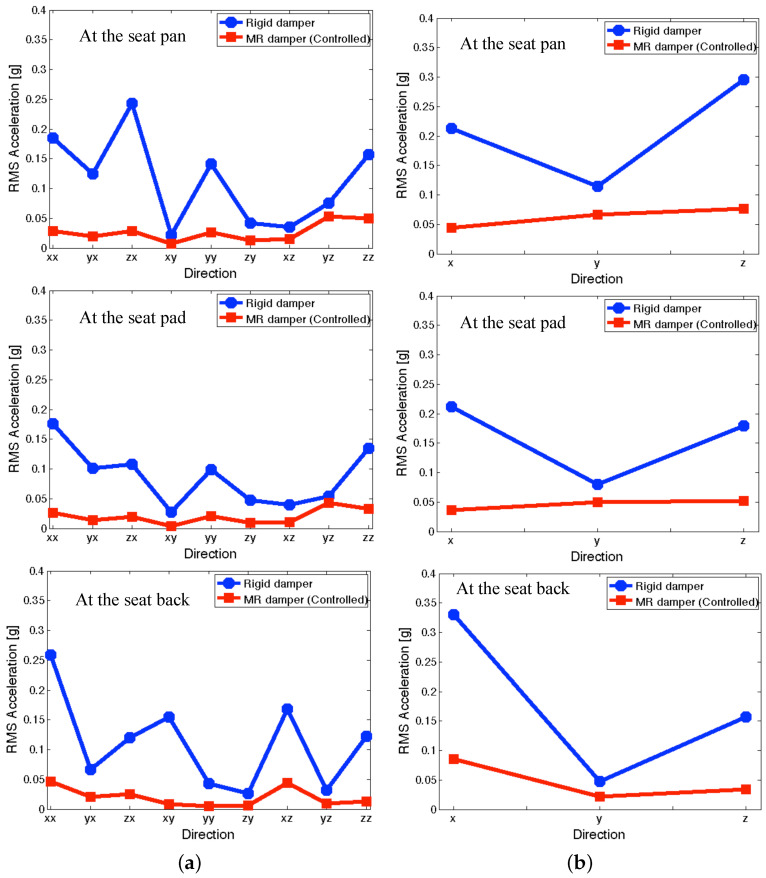
RMS accelerations of the full-scale MR seat suspension under the NFSSC algorithm (**a**) for each directional excitation input and (**b**) for simultaneous three-axis excitation inputs.

Figure 13 presents the RMS accelerations of the full-scale MR seat suspension under the NFSSC algorithm. In Figure 13a, the symbols xx, yx, and zx, etc., can be interpreted similar to the symbols in Figure 10. As seen in Figure 13a, the controlled MR seat damper case shows good vibration isolation performance at all measured positions for each directional excitation input. For observing the vibration isolation performance under simultaneous three-axis (i.e., *x*, *y*, and *z* axes) excitation inputs, Figure 13b was presented. Because of simultaneous three-axis excitation inputs, the output RMS acceleration levels shown in Figure 13b were increased than the individual input case shown in Figure 13a. But for the controlled MR seat damper case, the increment of the RMS acceleration levels is much less than in the rigid damper case. As observed in Figure 13b, the controlled MR damper case still showed good vibration isolation performance at the seat pan, seat pad, and seat back for simultaneous three-axis excitation inputs.

**Figure 14:**
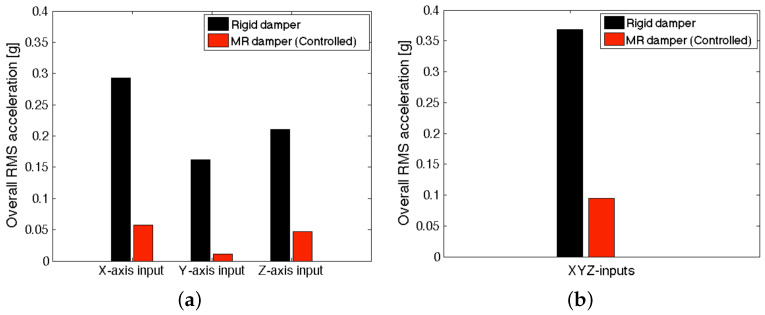
Overall RMS accelerations at the seatback of the full-scale MR seat suspension under the NFSSC algorithm (**a**) for each directional excitation input and (**b**) for simultaneous three-axis excitation inputs.

Since an occupant on a seat suspension will be exposed to all directional excitation inputs, we present the overall RMS accelerations at the seatback of the full-scale MR seat suspension under the NFSSC algorithm in Figure 14. In this case, the overall RMS accelerations, $a_{RMS}$, at the seatback were calculated by

(19) $$a_{RMS}=\sqrt{RMS{\left(a_{xk}\right)}^{2}+RMS{\left(a_{yk}\right)}^{2}+RMS{\left(a_{zk}\right)}^{2}}$$

Here, $a_{ik}$ are the output accelerations at the seatback, the subscript *i* is the output direction and the subscript *k* is the excitation input direction. As seen in Figure 14, the controlled MR seat damper case showed much lower overall MRS acceleration levels than the rigid damper case in all directions. For a single *X*-axis representative transient excitation input, the controlled MR seat damper case achieved an 80% seat vibration reduction improvement at the seatback position than the rigid damper case. For the *Y*- and *Z*-axis representative transient excitation inputs, the vibration reduction improvement of the controlled MR seat damper case was 93% and 78%, respectively. For simultaneous three-axis excitation inputs, the controlled MR seat damper case could make a 74% seat vibration reduction improvement.

## 5. Conclusions

In this study, the three-axis vibration isolation performance of a full-scale magnetorheological (MR) seat suspension under a narrow-band frequency domain semi-active (NFSSC) algorithm was experimentally investigated for both individual (in this case, excitation input was swept over a wider frequency range up to 200 Hz) and simultaneous three-axis directional inputs. From the results presented in this study, we reached the following conclusions:

1. From the single damper test on an MTS machine, it was experimentally observed that the three-axis MR seat damper used in this study could change the dynamic stiffness and loss angle significantly for both axial and lateral directional inputs.
2. From the single-degree-of-freedom (DOF) vibration isolation testing for the 1/3rd scale MR seat suspension, it was experimentally shown that the three-axis MR seat damper with the NFSSC algorithm can effectively reduce the axial seat vibration under a practical excitation vibration level of ±0.1 g. In addition, for the variations in the masses (i.e., 65 and 95 lbs), the MR seat damper with the NFSSC algorithm could still show good seat vibration isolation performance.
3. From the three-axis vibration isolation test for the full-scale MR seat suspension with a 50th percentile male dummy, it was experimentally proved that the controlled full-scale MR seat suspension could effectively suppress the three-axis seat vibrations for an individual sinusoidal excitation input. In addition, under both individual and simultaneous three-axis representative transient excitation inputs for military propeller aircraft, the controlled full-scale MR seat suspension showed a significant improvement in vibration isolation performance compared to the rigid seat suspension.
4. For individual representative transient excitation input, the controlled full-scale MR seat suspension could achieve a reduction in seat vibration at the seat back up to 93% than the rigid seat suspension. For simultaneous three-axis transient excitation inputs, the controlled full-scale MR seat suspension could improve 74% seat vibration reduction.
5. It was experimentally confirmed that the NFSSC algorithm could effectively suppress the seat vibration by using fewer sensors (for the vibration isolation testing of the full-scale MR seat suspension, only one three-axis accelerometer was required for the implementation of the NFSSC algorithm).

## Data Availability

Data available upon reasonable request.

## Abbreviations

The following abbreviations are used in this manuscript:

DOF	Degree of Freedom
DSP	Digital Signal Processing
MAST	Multi-Axis Simulation Table
MR	Magneto-Rheological
MTS	Material Testing System
NFSSC	Narrow-band Frequency Shaped Semi-Active Control
RMS	Root Mean Square
